# New Insights into the Genetic Diversity of *Clostridium botulinum* Group III through Extensive Genome Exploration

**DOI:** 10.3389/fmicb.2016.00757

**Published:** 2016-05-19

**Authors:** Cédric Woudstra, Caroline Le Maréchal, Rozenn Souillard, Marie-Hélène Bayon-Auboyer, Isabelle Mermoud, Denise Desoutter, Patrick Fach

**Affiliations:** ^1^Laboratory for Food Safety, French Agency for Food, Environmental and Occupational Health & Safety – Université Paris-EstMaisons-Alfort, France; ^2^Hygiene and Quality of Poultry and Pig Products Unit, Ploufragan-Plouzané Laboratory, UEB, French Agency for Food, Environmental and Occupational Health & SafetyPloufragan, France; ^3^l’UBL Université Bretagne LoireRennes, France; ^4^Avian and Rabbit Epidemiology and Welfare Unit, Ploufragan-Plouzané Laboratory, UEB, French Agency for Food, Environmental and Occupational Health & SafetyPloufragan, France; ^5^Laboratoire Public Conseil, Expertise et Analyse en BretagnePloufragan, France; ^6^Veterinary Diagnostic Laboratory, Laboratoires Officiels Vétérinaires, Agroalimentaires et Phytosanitaires, La Direction des Affaires VétérinairesPaïta, New Caledonia

**Keywords:** animal botulism, *Clostridium botulinum* group III, CRISPR, phage, whole genome sequencing

## Abstract

Animal botulism is caused by group III *Clostridium botulinum* strains producing type C and D toxins, or their chimeric forms C/D and D/C. Animal botulism is considered an emerging disease in Europe, notably in poultry production. Before our study, 14 genomes from different countries were available in the public database, but none were from France. In order to investigate the genetic relationship of French strains with different geographical areas and find new potential typing targets, 17 strains of *C. botulinum* group III were sequenced (16 from France and one from New Caledonia). Fourteen were type C/D strains isolated from chickens, ducks, guinea fowl and turkeys and three were type D/C strains isolated from cattle. The New Caledonian strain was a type D/C strain. Whole genome sequence analysis showed the French strains to be closely related to European strains from *C. botulinum* group III lineages Ia and Ib. The investigation of CRISPR sequences as genetic targets for differentiating strains in group III proved to be irrelevant for type C/D due to a deficient CRISPR/Cas mechanism, but not for type D/C. Conversely, the extrachromosomal elements of type C/D strains could be used to generate a genetic ID card. The highest level of discrimination was achieved with SNP core phylogeny, which allowed differentiation up to strain level and provide the most relevant information for genetic epidemiology studies and discrimination.

## Introduction

*Clostridium botulinum* is the aetiological agent of botulism, a deadly paralytic disease that can affect both humans and animals. The symptoms are caused by botulinum neurotoxins (BoNTs) typically produced by *C. botulinum*, a Gram-positive bacterium. The *C. botulinum* species can be divided into four groups (I to IV) based on physiological and genomic traits (Sobel, 2005). Animal botulism is mainly due to *C. botulinum* group III, which belongs to the *C. novyi sensu lato* species (Skarin et al., 2011). They produce toxin types C and D in addition to their mosaic variants C/D and D/C (Takeda et al., 2005). Animal botulism is considered an emerging disease in Europe, notably in poultry production (Souillard et al., 2014), where it can lead to significant economic losses (Lindberg et al., 2010). We previously showed that animal botulism in Europe is mainly due to mosaic type C/D in avian species, and type D/C in cattle (Woudstra et al., 2012). The development of new methods for the rapid molecular detection (Woudstra et al., 2012) and characterization (Anza et al., 2014; Woudstra et al., 2015a) of strains involved in outbreaks have so far revealed marked genetic similarity. This supports the hypothesis that one clade of closely related strains responsible for animal botulism dominates in Europe. This assumption is based on information gathered on strains isolated from outbreaks and typed using methods such as PFGE or flagellin gene detection by real-time PCR (Anza et al., 2014; Woudstra et al., 2015a). These methods have been universally used to check for strain diversity. However, the level of discrimination provided by these techniques may be insufficient to differentiate closely related genetic strains.

Since the arrival of next-generation sequencing technologies, the investigation of whole genome sequences of *C. botulinum* has given the scientific community invaluable new insights and provided genetic information of relevance for epidemiological investigations (Skarin and Segerman, 2014; Giordani et al., 2015; Weedmark et al., 2015a). At the time of our study, 14 genomes of *C. botulinum* group III were available in the public database: two of type C, seven of type C/D, three of type D, and two of type D/C. These genomes mostly originated from Sweden, and none were from France. Given this situation, our objective was to investigate *C. botulinum* group III genome sequences from France to complete the genetic picture of European *C. botulinum* group III strains and to find new potential genetic targets for typing. The genome sequences of 17 *C. botulinum* group III strains were analyzed: 14 type C/D strains from chickens, ducks, guinea fowl, and turkeys, plus three type D/C strains from cattle, all from France except for one type D/C strain which was from New Caledonia (Woudstra et al., 2015b). The sequences produced were processed and assembled either with a mapping or *de novo* process before being investigated for their genetic diversity using bio-informatic tools.

## Materials and Methods

### Isolates

**FIGURE 1:**
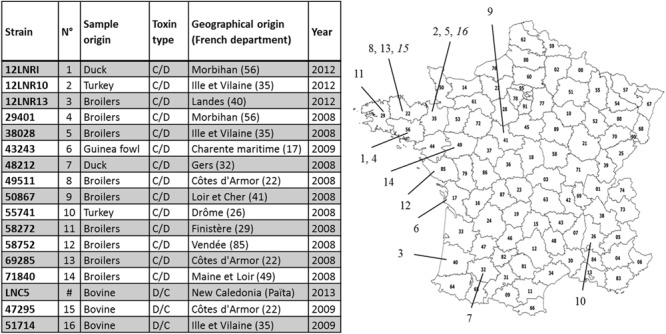
**Metadata for the isolated and sequenced *Clostridium botulinum* group III strains.** The numbers in brackets correspond to the French departmental code (geographical location of the outbreak associated with the strain recovered). They are indicated on the map of France. The location of the two French outbreaks of type D/C are indicated in italics (numbered 15 and 16).

All but one of the *C. botulinum* strains investigated in this study are from France. They were provided by LABOCEA in Brittany in the framework of Anses’s work as the French National Reference Laboratory for avian botulism. One isolate originated from New Caledonia following collaboration with the National Laboratory of New Caledonia. Isolates producing type C/D toxin mainly originated from chicken samples, whereas all BoNTs producing type D/C isolates were from bovine sample material. The samples were collected in different geographical areas, mainly from the north-western part of France, during botulism outbreaks that occurred between 2008 and 2013 (**Figure 1**).

### Culture Conditions, DNA Isolation, and Genome Sequencing

Genomic DNA was extracted from a 48-h culture incubated at 37°C under anaerobic conditions (A35 station, Don Whitley) in TPGY medium using the DNeasy blood and tissue kit (Qiagen, Hilden, Germany) according to the manufacturer’s instructions for Gram-positive bacteria, with an additional RNase A (Roche) treatment. Libraries were prepared using the Nextera XT kit (Illumina). Whole genome sequencing was performed using an Illumina MiSeq platform (Illumina) according to the manufacturer’s instructions. Three MiSeq runs were carried out, two with paired-end 150-nucleotide (nt) reads on MiSeq V2 and V2 microchemistry, another with paired-end 300-nt reads on V3 chemistry (Woudstra et al., 2015b).

### Nucleotide Sequence Accession Numbers

The sequence reads of these *C. botulinum* group III strains were deposited in the Sequence Read Archive (SRA) under accession numbers SRR2120181, SRR2124854, SRR2124859, SRR2124863, SRR2124865, SRR2124866, SRR2124867, SRR2124869, SRR2124870, SRR2124883, SRR2124884, SRR2124885, SRR2124886, SRR2124887, SRR2124888, SRR2124889, and SRR2124890.

### Genome Assembly

*De novo* assembly was performed as previously described (Woudstra et al., 2015b). Raw reads were trimmed (minimum length 35 bp, quality score 0.03) and assembled with the CLC Genomics Workbench version 7.5.1 (Qiagen, Aarhus, Denmark). The contigs created were ordered using *C. botulinum* C/D BKT015925 as a reference (GenBank accession number CP002411).

Mapping was performed using the NGS core “*Map reads to reference*” tools from CLC^®^ Genomics Workbench software version 7.5.1. Sequences of *C. botulinum* group III type C/D strains were mapped using *C. botulinum* type C/D BKT015925 as a reference (GenBank accession number CP002411). Mapping sequences were considered successful only if the depth was more than 10X. Over 30X was considered a good sequencing depth (Ekblom and Wolf, 2014; Sims et al., 2014). As no reference sequence was available for type D/C, *C. botulinum* group III type D/C isolates were mapped using draft genome CCUG7971 type D as a reference for strain LNC5 and draft genome DC5 type D/C for strains 47295 and 51714.

### Comparative Genome Analyses

Gegenees software (Agren et al., 2012; version 2.2.1^1^), a comparative analysis tool for whole genome sequence data, was used to provide a phylogenetic overview of the microbial genome sequences investigated. The software uses a fragmented approach to make all-against-all BLASTN whole genome comparisons. A fragment length of 200 bp and step size of 100 bp were used. Dendrograms were produced in SplitsTree4 (Huson and Bryant, 2006; version 4.13.1^2^) using the neighbor-joining method from a Nexus file exported from Gegenees. BLAST Ring Image Generator (BRIG) software (Alikhan et al., 2011; version 0.95^3^) was used to further compare the genome sequences.

### Core SNP Phylogeny Profiling

*De novo*-assembled contigs of the French *C. botulinum* group III isolates and publicly available group III genome sequences produced by Skarin and Segerman (2014) were submitted to the CSI Phylogeny 1.1 web server^4^ for analyses. This server identifies SNPs from whole genome sequencing data, filters and validates the SNP positions, and then infers phylogeny based on concatenated SNP profiles (Kaas et al., 2014). *C. botulinum* BKT015925 type C/D was used as the reference strain. SNPs were excluded if they were in regions with a minimum fold coverage of <10, within 10 bp of another SNP or <15 bp from the end of a contig. A maximum likelihood tree was created from the concatenated SNP sequences and images were rendered in FigTree (v1.4.1^5^).

### Phage Sequence Search

The phage search tool web server (Zhou et al., 2011; PHAST^6^), designed to rapidly and accurately identify, annotate and graphically display prophage sequences within bacterial genomes or plasmids, was used to investigate the sequences produced.

### CRISPR Identification

CRISPR–Cas genes were identified using PROKKA annotation pipeline (Seemann, 2014). The CRISPR recognition tool software (Bland et al., 2007) version 1.0 was used to investigate sequences for clustered regularly interspaced short palindromic repeats. The settings were chosen to search for a minimum number of three repeats with a minimum repeat length of 19 nucleotides and a maximum repeat length of 38 nucleotides, within a search window of eight. Spacer settings were for a minimum length of 19 nucleotides and a maximum of 48.

## Results

### Assemblies and Genome Sequences Data

The genomes of the 14 strains of *C. botulinum* group III type C/D and the three *C. botulinum* type D/C strains were *de novo*-assembled. A table containing the information related to assembly statistics of the sequences produced was previously published (Woudstra et al., 2015b). The sequences were successfully checked to meet the requirements of genome quality for Illumina technology (Mavromatis et al., 2012). Genome size, GC content, and the number of coding sequences were concordant with reference strains *C. botulinum* type C/D BKT015925 (Skarin et al., 2011). Mapping assembly was also performed using reference genome BKT015925 for type C/D strains (**Table 1**). The analysis of the mapped sequences show that plasmid p1 containing the botulinum locus and plasmids p2 and p3 are always associated with-type C/D isolates. The sequence depth associated with plasmid p1 shows that isolates 12LNRI, 58752, and 69285 were not sequenced deeply enough (respectively, 22X, 10X, and 5X). This could be because there are fewer prophage copies in the strains than chromosome copies. This is an illustration of the pseudolysogeny phenomenon, the prophage being known to be unstable during cultivation (Oguma, 1976). Plasmids p4 and p5 were not systematically present. P4 was found in strains 38028, 48212, 50867, 58272, and 69285 but absent in the other strains. P5 was found in all the strains except 12LNR13, 49511, and 69285. Regarding type D/C sequences, in the absence of a complete reference genome for *C. botulinum* type D/C, strains 47295, 51714, and LNC5 were mapped against the most genetically closely related draft genomes available, meaning CCUG7971 type D for LNC5 and DC5 type D/C for strains 47295 and 51714. Plasmids p1, p2, and p3 were identified, but no sequences related to plasmid p4 and p5 were retrieved. These results highlight the genetic difference between *C. botulinum* type C/D and type D/C.

**Table 1:** *Clostridium botulinum* type C/D mapping assembly details.

Strain	Origin	Mapping	Chr	p1	p2	p3	p4	p5
12LNRI	Duck	Coverage	98%	98%	98%	95%	2%	100%
		Depth (X)	90	23	193	195	0	143
12LNR10	Turkey	Coverage	98%	98%	98%	95%	6%	100%
		Depth (X)	88	151	119	146	8	439
12LNR13	Chicken	Coverage	96%	88%	85%	90%	0%	17%
		Depth (X)	65	57	58	86	0	0
29401	Chicken	Coverage	98%	98%	99%	95%	5%	100%
		Depth (X)	101	120	83	118	6	426
38028	Chicken	Coverage	96%	82%	97%	91%	82%	72%
		Depth (X)	92	97	97	124	183	419
43243	Guinea fowl	Coverage	98%	98%	97%	95%	0%	100%
		Depth (X)	69	57	57	91	0	261
48212	Duck	Coverage	97%	98%	97%	94%	100%	100%
		Depth (X)	88	88	101	134	66	374
49511	Chicken	Coverage	95%	91%	90%	91%	6%	1%
		Depth (X)	46	48	42	60	1	0
50867	Chicken	Coverage	97%	98%	98%	96%	100%	100%
		Depth (X)	55	30	110	135	97	519
55741	Turkey	Coverage	98%	99%	98%	96%	5%	100%
		Depth (X)	95	91	65	102	4	68
58272	Chicken	Coverage	97%	98%	98%	94%	95%	100%
		Depth (X)	72	104	131	97	94	223
58752	Chicken	Coverage	97%	93%	98%	95%	7%	100%
		Depth (X)	99	10	171	212	15	775
69285	Chicken	Coverage	98%	94%	98%	96%	55%	9%
		Depth (X)	76	5	93	142	399	0
71840	Chicken	Coverage	97%	98%	97%	94%	0%	100%
		Depth (X)	44	43	48	61	0	241

*All strains belong to *C. botulinum* group III type C/D. The chromosome sequence (Chr) is 2773157 bp long; p1 corresponding to the botulinum prophage, contains botulinum and C3 toxin, and is 203287 bp long; p2 contains alpha-toxin and is 98732 bp long; p3 contains C2 toxin and phospholipase C, and is 80365 bp long; p4 contains phage sequence phiCD6356 (Horgan et al., 2010) and is 39648 bp long; p5 contains *C. perfringens* putative epsilon toxin (Skarin and Segerman, 2014) and is 12403 bp long.*

The unused reads from the mapping assembly of type C/D and D/C isolates were collected and *de novo*-assembled to search for genetic elements that differ from the reference genomes (data not shown). The results show that for some isolates, *de novo* assembly of unused mapping sequences gathers into contigs long enough to be considered true genetic elements. Strains 12LNRI, 43243, 69285, 71840, 47295, and 51714 produced relatively short contig assemblies between 1300 and 8300 bp, which were related to 16S and 23S RNAs (data not shown). The other isolates gave contigs ranging up to 45 kbp, reflecting the presence of large genetic sequences that may be related to extrachromosomal elements. This emphasizes the benefits of investigating unmapped reads while doing resequencing analyses (Gouin et al., 2015).

### Comparative Genome Analyses

**FIGURE 2:**
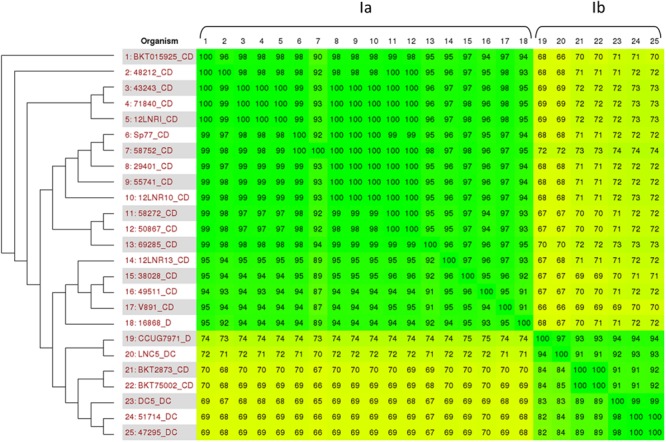
**Comparison between *C. botulinum* type C/D and D/C genome sequences and *C. novyi sensu lato*.** Based on the work of Skarin and Segerman (2014) and the publicly available genome sequences, the genomes investigated were analyzed using Gegenees software. The comparison matrix obtained clearly associated our genomes with *C. novyi sensu lato* group Ia and Ib species.

As previously published by Skarin and Segerman (2014), *C. botulinum* group III is part of the larger group *C. novyi sensu lato* which also encompasses *C. novyi* and *C. haemolyticum*. It appears to be divided into four different lineages, with *C. botulinum* type C/D and type D/C mainly clustering into lineage Ia and Ib, respectively. **Figure 2** represents the phylogenetic relationship between the *de novo*-assembled genome sequences of French *C. botulinum* group III and the publicly available *C. novyi sensu lato* genome sequences of lineage Ia and Ib, using Gegenees matrix comparison. The French type C/D isolates are clearly associated with group Ia, while those of type D/C are related to group Ib. None of the French isolates investigated were associated to *C. novyi* or *C. haemolyticum*.

The comparison matrix produced using BKT015925 as a reference value clearly distinguishes *C. botulinum* types C/D and D/C, with an average sequence identity of 70%. BKT015925 was shown to be genetically closely related to the French C/D isolates (94–100% identity). Twelve of these isolates showed 99–100% sequence identity with BKT015925, emphasizing their close genetic relationship. Strains 38028, 12LNR13, and 49511 could be considered more distantly related to BKT015925 (95% genetic identity) and to the other French isolates (87–96%). Alignment of these sequences with BKT015925 using BRIG software show that isolates 38028, 12LNR13, and 49511 lack a specific region of 16 kbp (data not shown). This missing region contains eight coding sequences (CbC4-2081 to CbC4-2088) that encode five hypothetical proteins and three proteins known to be involved in phosphorothioation DNA modification (DndD, DndE and a cysteine desulfurase). This mechanism modifies the DNA backbone, with sulfur replacing non-bridging phosphate oxygen (Wang et al., 2007). It was found to be responsible for the DNA degradation phenotype that renders modified DNA susceptible to cleavage during PFGE migration runs (Zhou et al., 2005; Ho et al., 2015). The intact genetic locus comprises five Dnd genes (DndA/B/C/D/E) and a regulator gene, IscS (that could be replaced by a cysteine desulfurase). A recent investigation has shown that DndA is not indispensable in *Streptomyces lividans*, but all the other Dnd genes are necessary for the locus to be functional (Xu et al., 2009). Thus, strains 38028, 12LNR13, and 49511—which lack DndD, DndE and the cysteine desulfurase—should have a dysfunctional DNA phosphorothioation mechanism and thus be able to be typed by PFGE, while other strains containing the intact Dnd locus would probably not be typeable. Investigation of the presence of this Dnd locus in strains from Skarin et al. (2011) study showed BKT015925, Sp77 and V891 to be Dnd positive, while strains 16868, CCUG7978, DC5, BKT75002, and BKT2873 were negative. Such a phenotype could explain the PFGE typing difficulties encountered with certain *C. botulinum* group III strains (Anza et al., 2014).

Genome sequences of *C. botulinum* type D/C (strains 47295, 51714, and LNC5) were observed to belong to cluster Ib (Skarin and Segerman, 2014). Isolates 47295 and 51714 were highly genetically related to strain DC5 type D/C originating from Italy. On another hand, isolate LNC5 was more closely related to strain CCUG7971 type D isolated in South Africa. The whole genome size of strains LNC5 and CCUG7971 were smaller than other type D/C strains (2.89 and 2.81 Mbp, respectively, compared to ≥3.18 Mbp); it may thus be hypothesized that this could reflect differences in the content of mobile genetic elements. A closed reference genome of *C. botulinum* type D/C and further thorough analyses of more genome sequences would be necessary to fully investigate the genetic relatedness of type D/C strains.

### SNP Profiling

To determine whether closely related isolates could be resolved by outbreak or region of origin, SNP profiling was performed on both French *C. botulinum* group III isolates and publicly available genome sequences (Skarin and Segerman, 2014). The botulinum locus sequences (botR to *bont*/C, 11575 bp) were investigated for SNPs. All the isolates investigated harbor the same ha+ locus sequence. BotR, HA17, HA70, HA33, ntnh, and *bont* sequences are identical to the BKT015925 botulinum locus, except for isolates 58752 and 69285 for which the locus was not totally covered (missing 574 and 908 bp, respectively). This could be the consequence of the poor depth coverage of plasmid p1 for these two isolates (respectively, 10X and 5X). Only four SNPs were retrieved on the entire botulinum locus, all identical for strains 49511, 12LNR13, and 38028 type C/D. Two are located in gene HA70 and the other two in gene HA17 (position 2395 G-A, 2457 T-C, 2529 C-T, and 2575 T-C). This is in accordance with the results of the whole genome comparison made with Gegenees software (see **Figure 2**) that showed strains 49511, 12LNR13, and 38028 to cluster slightly differently from other type C/D strains.

**FIGURE 3:**
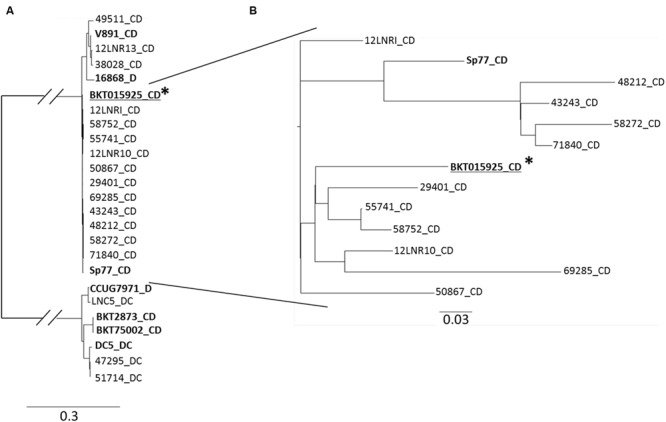
***C. botulinum* group III SNP phylogeny profiling. (A)** Maximum likelihood tree created from concatenated SNPs sequences using CSI phylogeny webserver 1.1 (https://cge.cbs.dtu.dk/services/CSIPhylogeny; Kaas et al., 2014). *C. botulinum* group III genome sequences from Skarin and Segerman (2014) are bolded. ^∗^*C. botulinum* type C/D reference sequence used. **(B)** Highly genetically related French *C. botulinum* strains Group III type C/D isolates were analyzed separately to solve a better discriminatory level.

Core SNP profiling of both the French type C/D and D/C strains and the strains from the Skarin and Segerman (2014) study, were investigated using the CSI Phylogeny web server of the Centre for Genomic Epidemiology of DTU in Denmark (Kaas et al., 2014) (**Figure 3**). The reference sequence used was *C. botulinum* type C/D BKT015925. The SNP tree distinguishes three major groups (**Figure 3A**). The results showed a clear distinction between the French type C/D strains 12LNR13, 38028, and 49511 which clustered together with strain V891 type C/D (from Sweden) and strain 16868 type D (from The Netherlands), and remained distant from the other isolates. All the other French type C/D strains clustered together with the reference strain BKT015925 and Sp77 type C/D (originating from Sweden and Spain, respectively). As expected, *C. botulinum* type C/D isolates clustered together at a distance from type D/C isolates, which is in accordance with previous results. The LNC5 type D/C and CCUG7971 type D strains clustered together, while strains 47295, 51714, and DC5 type D/C clustered separately, which is in accordance with the Gegenees matrix comparison results.

To further enhance discrimination, we focused on the genetically closely related French *C. botulinum* group III type C/D isolates (**Figure 3B**). French strains 48212/43243/58272/71840 and to a lesser extent 12LNRI, were related to strain Sp77 (originating from Spain). Strains 29401/55741/58752/12LNR10/69285 and to a lesser extent strain 50867 were related to reference strain BKT015925 (originating from Sweden). The results showed no correlation between toxin type, geographical and source origin. Despite their marked genetic degree of relatedness, SNP core phylogeny was able to clearly differentiate the French *C. botulinum* group III strains investigated even up to the outbreak origin.

### Comparative Phage Typing

The SNP core phylogeny proved to be highly discriminant but requires the analysis of whole genome sequences. Although, nowadays benchtop sequencers are more affordable and easy to use, the analysis of extensive sequencing data produced still presents a challenge and prevents the routine application of WGS in veterinary laboratories. Another issue to develop this approach is the relative high difficulty to get *C. botulinum* group III isolated. Thus, there is a need to develop typing methods that could reach the same level of discrimination without the need of a whole genome sequence. The previous work of Skarin and Segerman (2011) showed *C. botulinum* group III to contain several phage sequences. We postulate it would be possible to use the mobile genetic element content of *C. botulinum* group III for typing purposes. We searched for the presence of extrachromosomal elements differing from reference strain BKT015925 using the PHAST web server (Zhou et al., 2011). PHAST identified three family phage sequences—phiSM101, phiS63, and phi3626—which had never previously been characterized in *C. botulinum* group III. Using this information, together with the presence of BKT015925 plasmids and phages, it was then possible to create a genetic ID card of the mobile genetic elements present in the investigated isolates (**Table 2**). Regarding type C/D, isolate 48212 contains the same mobile element as reference strain BKT015925. Isolates 50867 and 58272 also contain the same five mobile elements as BKT015925, plus a sixth one which is attributed to phage sequence family phiSM101, a 38-kbp phage encountered in *C. perfringens* (Myers et al., 2006). Strain 38028 contains the five mobile elements of BKT015925, plus phiSM101 sequences and a seventh attributed phage sequence, phi3626, a bacteriophage of 33.5 kbp characterized in *C. perfringens* (Zimmer et al., 2002). Strains 12LNR10, 29401, 55741, and 58752 lack the plasmid p4 sequence [phiCD6356, encountered in *C. difficile* (Horgan et al., 2010)] from BKT015925, but harbor phiSM101. Isolate 49511 lacks elements phi6356 and plasmid p5, but contains phiSM101. Strains 12LNRI, 43243, and 71840 lack phiCD6356 element from BKT015925. Strain 12LNR13 lacks sequences phiCD6356 and p5 from BKT015925, but contains a sequence attributed to phiS63, a 33.6-kbp phage from *C. perfringens* not found in any other isolates. Finally, strain 69285 lacks phiCD6356 and p5 from BKT015925 and does not contain any other mobile genetic elements. Thus, using the presence/absence of mobile genetic element sequences in *C. botulinum* type C/D isolates, it is possible to differentiate eight groups, which constitutes a non-negligible improvement in the typing of these strains compared to previously developed flagellin typing method (Woudstra et al., 2015a). The search for extrachromosomal elements in type D/C isolates showed them to contain sequences related to p1, p2, and p3. Isolates 47295 and 51714 contain also phiSM101, whereas LNC5 does not, and no other phage related sequences except for the botulinum prophage. It was quite surprising to see such difference in the mobilome content between type C/D and type D/C isolates. We thus asked ourselves the reason of such difference. We hypothesized that some mechanism regulating the presence or absence of extrachromosomal elements could be incriminated, such as the CRISPR–Cas (clustered regularly interspaced short palindromic repeats–CRISPR-associated proteins) system.

**Table 2:** *C. botulinum* type C/D genetic mobile element ID card.

							Phage attributed sequence			
Strain	Origin	p1	p2	p3	p4	p5	phiSM101	phiS63	phi3626	Phage group
BKT015925	Chicken	+	+	+	+	+	-	-	-	I
48212	Duck	+	+	+	+	+	-	-	-	I
12LNRI	Duck	+	+	+	-	+	-	-	-	II
43243	Guinea fowl	+	+	+	-	+	-	-	-	II
71840	Chicken	+	+	+	-	+	-	-	-	II
50867	Chicken	+	+	+	+	+	+	-	-	III
58272	Chicken	+	+	+	+	+	+	-	-	III
38028	Chicken	+	+	+	+	+	+	-	+	IV
12LNR10	Turkey	+	+	+	-	+	+	-	-	V
29401	Chicken	+	+	+	-	+	+	-	-	V
55741	Turkey	+	+	+	-	+	+	-	-	V
58752	Chicken	+	+	+	-	+	+	-	-	V
12LNR13	Chicken	+	+	+	-	-	-	+	-	VI
69285	Chicken	+	+	+	-	-	-	-	-	VII
49511	Chicken	+	+	+	-	-	+	-	+	VIII

*C. botulinum type C/D strains are grouped according to their mobile genetic content. P1 contains botulinum and C3 toxin, p2 contains alpha-toxin, p3 contains C2 toxin and phospholipase C, p4 contains phage sequences attributed to phiCD6356 and finally p5 contains *C. perfringens* putative epsilon toxin (Skarin and Segerman, 2014). PHAST web server-attributed phage sequences (phi SM101, phiS63, phi3626) were identified.*

### Comparative CRISPR Analyses

The CRISPR–Cas mechanism is an adaptive immunity system that is present in many archaea and bacteria and which act against invading genetic elements. It consists of distinct arrays of short repeats interspersed with unique spacer’s content, with Cas proteins encoded by putative operons adjacent to CRISPR sequences. One hypothesis to explain the diversity of extrachromosomal elements encountered in type C/D strains and the absence of such elements in type D/C, would be the CRISPR loci to be different between them. Analysis of the CRISPR–Cas sequences of the reference genome *C. botulinum* type C/D BKT015925, showed to contain CRISPR–Cas genes Cas3/Cas5 (located between the IS transposase proteins) and Cas3/Cas5/Cas6, present on the prophage (p1) and on plasmid p2, respectively (Skarin et al., 2011). Based on a review by Makarova et al. (2011), we could assume that these CRISPR loci belong to CRISPR type I-B and are incomplete. It therefore could be hypothesized to be non-functional for BKT015925 type C/D. Investigation of the genome sequences for the French type C/D strains revealed that CRISPR loci were of type I-B also and located on plasmid p2. It contains Cas3/Cas5/Cas6/Cas7 and a homolog gene to Cas8b1, lacking Cas1/Cas2 and Cas4 genes. Cas1 is involved in the integration of spacer DNA into CRISPR repeats, Cas2 facilitates spacer selection and/or integration, and Cas4 might be involved in spacer acquisition (Makarova et al., 2011). We can therefore suggest that this CRISPR locus is deficient in integrating new foreign DNA. The CRISPR-associated (Cas) proteins that provide acquired immunity against foreign DNA are inactive in type C/D strains, which could explain the presence of so many extrachromosomal elements. On the contrary, type D/C strains contain a fully functional type I-B CRISPR system and a partially deficient type III-B system (lacking cmr1 and cmr3 genes, which could be involved in RNA-guided RNA cleavage), chromosomally located. It was furthermore possible to identify a fully functional CRISPR sequence related to system I-D, located on plasmid p2. Type D/C strains were shown to contain two active CRISPR systems and one potentially inactive system; this would explain why phage elements were so scarce in the genome of the type D/C strains investigated.

CRISPR regions are also known in other bacteria to be an appropriate target for high-resolution strain typing (Delannoy et al., 2012), the spacer sequences being representative of the historical encounter of the bacteria with extrachromosomal mobile genetic elements. As we were not able to use the mobilome content in type D/C strains as typing targets, we investigated the CRISPR sequences diversity to achieve typing resolution. Isolates 47295 and 51714, which were highly genetically related with strain DC5 from Italy, showed their CRISPR content to be quite different, with only two common CRISPR loci. It was also possible to differentiate between strain 47295 and 51714 by one CRISPR loci. Based on their genome sequences comparison we could hypothesize that strains 47295, 51714, and DC5 are closely related. The analysis of their CRISPR content confirms this assumption but could also help differentiate them. Also, CRISPR sequences of strain LNC5 type D/C was observed to differ greatly from that of strains 47295 and 51714 with only one CRISPR locus that could be related to strain 51714, all other CRISPR sequences being totally unrelated, supporting the hypothesis that strain LNC5 is of a different origin. Although strain LNC5 was closely related to strain CCUG7971 from South Africa, they share only one CRISPR loci. On another hand, investigation of French type C/D isolates for their CRISPR genetic diversity revealed it to be not discriminatory enough to differentiate between them, as expected due to the presence of a deficient CRISPR–Cas mechanism.

## Discussion

Seventeen isolates of *C. botulinum* group III types C/D and D/C were characterized *in silico* using whole genome sequence data. No genome data from French *C. botulinum* group III strains was available beforehand. The previous work of Skarin and Segerman (2014) was the first to investigate and to grant insights into the genomics of *C. botulinum* group III. It pointed out the high degree of genetic relatedness between isolates from different sources and geographical origins. The present work investigated French isolates in order to shed light on the genomic diversity of *C. botulinum* group III. The analyses confirmed marked similarity among the investigated strains and the publicly available genome sequences originating from Europe, clustering French strains into lineage Ia and Ib. This had already been reported when comparing strains from Spain and Sweden (Anza et al., 2014). No correlation was observed between geographical origin and source of the strains. It was possible to clearly discriminate type C/D from type D/C. Interestingly, there was a marked genetic correlation between strain LNC5 type D/C, isolated from cattle, and historical strain CCUG7971 type D, isolated in South Africa in 1929. This appears to show that LNC5 is more closely related to CCUG7971—despite being a type D/C and not D strain like CCUG7971—than to European type D/C strains. It might be interesting to compare group III *C. botulinum* from parts of the world outside Europe to investigate such potential geographical differences. Our investigation also revealed genomic diversity into the Dnd locus responsible for phosphorothioation DNA modification of the DNA backbone, with sulfur replacing non-bridging phosphate oxygen, and being responsible for a degradation pattern observed during PFGE electrophoresis. It is hypothesized that endowment of DNA with sulfur gives it an oxidation-reducing chemical property that protects the hosting bacteria against peroxide (Xie et al., 2012). Therefore, bacteria lacking this functional mechanism would be typeable by PFGE, but probably more sensitive to oxidative stress.

A huge rise in the number of avian botulism outbreaks in poultry production was observed in France between 2007 and 2011. The origin of this upsurge is typically unknown. Gegenees and SNP analysis show that strains 49511, 38028, and 12LNR13 differ from the other type C/D strains studied here. These strains were isolated from broiler outbreaks in 2008 and 2012. This result seems to show that at least two groups of type C/D strains were involved in poultry botulism outbreaks between 2008 and 2012 in France. Interestingly, BKT015925—which is very similar to 12 C/D strains included in our study—was also isolated in 2008 from broilers in Sweden. Strain V891, which is very similar and related to strains 49511, 38028, and 12LNR13, was isolated in 2007 from a wild herring gull that had died from botulism in Sweden. This result implies that at least two groups of *C. botulinum* type C/D strains are involved in avian botulism outbreaks in Europe. It appears that there is no correlation between avian species and the type C/D group, but this tends to demonstrate that the same strains are involved in poultry and wild bird outbreaks. It is now necessary to analyze the genome of strains isolated from botulism outbreaks in wild birds to confirm this initial observation.

More detailed investigations of mapping assembly sequences, and particularly the unmapped reads, using the PHAST web server, revealed new genetic elements related to phage sequences in type C/D strains. These results helped discriminate closely related isolates into eight different groups. Interestingly, type D/C strains showed to harbor few mobile elements in comparison. The presence of new mobile genetic elements, which could be phage related, is in accordance with the large plasmidome content already characterized in *C. novyi sensu lato* (Skarin and Segerman, 2014). Therefore, by using specific genetic targets for each plasmid or phage sequence, it may be possible to generate an ID card, which would constitute an improvement in typing development. Yet this method only constitutes a pre-screening step that would be applicable only to type C/D strains. While investigating the extrachromosomal content we showed that the difference in the mobilome content between type C/D and type D/C strains could be attributed to a deficient CRISPR–Cas mechanism in strains type C/D. The investigation of CRISPR sequences diversity in type D/C strains for typing purposes helped differentiating between highly genetically related strains, while it did not improve matters in discriminating between type C/D strains. In conclusion, the mobilome content could be used for typing C/D strains, whereas CRISPR loci would be preferable to differentiate between type D/C strains.

*In silico* MLST could also be an option to investigate the genetic diversity of *C. botulinum* group III. The latest work of Weedmark et al. (2015b) investigated the possibility of using whole genome sequence data to resolve phylogenetic profiling of *C. botulinum* group II by outbreak or location of origin. A preliminary *in silico* test of the French *C. botulinum* group III genome sequences produced showed it was only possible to discriminate between type C/D and type D/C strains using Weedmark’s MLST-12 pattern (data not shown). This finding is not surprising as *C. botulinum* group III is genetically distantly related to groups I and II. It does not mean the method is irrelevant, but it will have to be adapted by defining specific locus targets for *C. botulinum* group III.

Regarding SNP genotyping, the phylogenetic trees obtained while investigating the French type C/D and D/C strains, compared with the closest publicly available genome sequences, allow three major groups to be differentiated. The French type D/C strain was shown to be very distant from type C/D, which may imply a different historical origin. The French type C/D strains were closely related, which corroborates the hypothesis of a clonal spread throughout Europe. Yet using SNP core phylogeny, it was possible to achieve the deepest characterization level, resolving strains by their outbreak origins. It is now necessary to sequence strains isolated from other countries and other outbreaks to identify discriminating SNPs that could be used for routine genotyping analysis. This opens up new avenues for epidemiological investigations and will be particularly useful for identifying sources of outbreaks.

## Conclusion

The comprehensive analyses of phage, plasmid, CRISPR, and SNP content revealed significant differences between strains of *C. botulinum* group III, showing plasticity in both the chromosomal core and mobilome of these strains, providing discriminative information for future genetic studies. In the light of the results presented here, we may speculate these strains to have probably different capacity to adapt and evolve in new environments.

## Author Contributions

Conceived and designed the experiments: CW and PF. Performed the experiments: CW. Analyzed the data: CW. Contributed reagents/materials/analysis tools: CW, CL, RS, M-HB-A, IM, DD, and PF. Wrote the paper: CW and PF. Critical revision of the paper for important intellectual content: CW, CL, RS, M-HB-A, IM, DD, and PF.

## Conflict of Interest Statement

The authors declare that the research was conducted in the absence of any commercial or financial relationships that could be construed as a potential conflict of interest.

## Footnotes

1 http://www.gegenees.org

2 http://www.splitstree.org/

3 http://brig.sourceforge.net/

4 https://cge.cbs.dtu.dk/services/CSIPhylogeny/

5 http://tree.bio.ed.ac.uk/software/figtree

6 http://phast.wishartlab.com/
